# Biomarkers/Molecular Targets, Immunotherapy, and Treatments for Non–Small Cell Lung Cancer

**DOI:** 10.6004/jadpro.2016.7.5.4

**Published:** 2016-07-01

**Authors:** Elizabeth S. Waxman, Frank V. Fossella

**Affiliations:** MD Anderson Cancer Center, Houston, Texas

## Abstract

For decades, the prognosis for patients with advanced-stage non–small cell lung cancer (NSCLC) was bleak, with chemotherapy offering limited benefit and much toxicity. Now, with mutational testing, new generations of targeted therapies, and emerging immunotherapies, the treatment horizon for these patients has greatly expanded. In this article, the authors review molecular targets, biomarkers, as well as immune checkpoint inhibitors, which are having a major impact on the management of this patient population.

Molecular targets, biomarkers, their treatments, and immunotherapy have changed the treatment paradigm for non–small cell lung cancer (NSCLC) from chemotherapy ("one-size-fits-all") approach to specific recommendations for patients based on the presence or absence of gene mutations ("personalized medicine"). For decades, chemotherapy—with modest response rates, survival rates measured in weeks/months, and significant toxicities—was the mainstay of treatment for patients with advanced-stage lung cancer. The current trend is to treat patients based on specific pathology (squamous cell carcinoma or nonsquamous cell carcinoma), and the presence (or not) of gene mutations.

Biomarkers have various functions including diagnostic, monitoring, staging, predictive, and prognostic values (Grande, Viale, & Yamamoto, 2010). Predictive markers determine the particular therapy for select patients (Aggarwal, Somaiah, & Simon, 2010; Grande et al., 2010). Prognostic markers forecast those tumors that are likely to recur (lead to death) regardless of therapy (Kreamer, Eaby-Sandy, Sherry, & Stonehouse-Lee, 2011).

Somatic genome alterations, known as "driver mutations," are the most useful predictive markers for determining the efficacy of targeted therapy (Sequist & Neal, 2015). Driver mutations are usually transformative, meaning they initiate the change from a noncancerous cell to a malignant cell (Sequist & Neal, 2015). Driver mutations pass on a reliance (oncogene addiction) on cancer cells to continuously receive signals from the driver to survive (signal transduction; Sequist & Neal, 2015). Normal cellular mechanisms, which regulate cell growth, differentiation, and cell death, no longer function. Epidermal growth factor receptor (EGFR), Kirsten rat sarcoma viral oncogene homolog (KRAS), anaplastic lymphoma kinase (ALK), and ROS1 are driver mutations.

## EPIDERMAL GROWTH FACTOR RECEPTOR

Epidermal growth factor receptor (EGFR) is the most common driver mutation in NSCLC, specifically adenocarcinomas (Lynch et al., 2004; Paez et al., 2004; Pao et al., 2004). This mutation belongs to the HER/ErbB family of receptor tyrosine kinases, which includes EGFR 2 (HER2/*neu*/ErbB2), EGFR 3 (HER3/ErbB3), and EGFR 4 (HER4/ErbB4; da Cunha Santos, Shepherd, & Tsao, 2011). Found on normal cells, EGFR is a transmembrane (has both extracellular and intracellular components), ligand-binding receptor (Kreamer et al., 2011). In normal cellular functions and pathways, EGFR has a significant role in cellular proliferation and differentiation (Yano et al., 2003).

During normal cellular activity, dimerization (ligands binding to extracellular receptors) and autophosphorylation occur, thus initiating an intracellular cascade of downstream signals resulting in normal cell growth, differentiation, and cell death (Kreamer et al., 2011). In malignant cells, dysregulation of the intracellular (tyrosine kinase) activity of EGFR may be caused by EGFR protein overexpression, *EGFR* gene mutations, and/or increased gene copy number (da Cunha Santos et al., 2011; Ciradello & Tortora, 2008), resulting in uncontrolled cellular proliferation, invasion, and inhibition of apoptosis (Kreamer et al., 2011).

***EGFR* Mutations**

Mutations in *EGFR* occur in approximately 15% of white and African American patients with NSCLC; 30% of NSCLC of Asian ethnicity; and are associated with adenocarcinoma histology, female gender, and nonsmoking status (Massarelli et al., 2013; Cote et al., 2011; Reinersman et al., 2011; Shigematsu et al., 2005; Tokumo et al., 2005). Mutations in *EGFR* exist in the first four exons (18–21) of the tyrosine kinase domain of EGFR (See Table; Kreamer et al., 2011). The most common mutations involve point mutations in exon 18, insertions or deletions in exon 19, insertions/duplications and point mutations in exon 20, and point mutations in exon 21 (Massarelli et al., 2013). Point mutations in exon 18, predominantly G719, account for approximately 4% to 5% of *EGFR* mutations and are less sensitive to EGFR tyrosine kinase inhibitors (TKIs; Massarelli et al., 2013; Sharma, Bell, Settleman, & Haber, 2007).

**Table:**
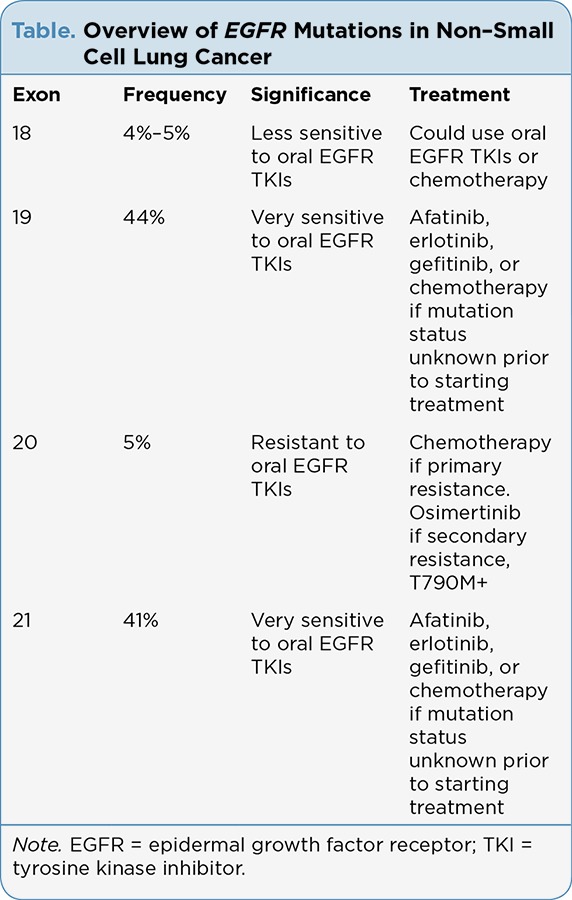
Overview of EGFR Mutations in Non–Small Cell Lung Cancer

The most common *EGF*R mutations are in exons 19 and 21 and account for 44% and 41% of all *EGFR* activation mutations, respectively, and are sensitive to treatment with EGFR TKIs (Massarelli et al., 2013). Mutations in *EGFR* in exon 19 include in-frame deletions, which frequently encompass L747 and E749; L858R is the most common point mutation for exon 21 (Massarelli et al., 2013). In-frame insertions and point mutations in exon 20 account for 5% of *EGFR* mutations (Zhang, Stiegler, Boggon, Kobayashi, & Halmos, 2010) and are resistant to EGFR TKIs (Massarelli et al., 2013).

**Treatment of *EGFR*-Mutant Disease**

Many factors are involved in treatment decisions for patients with NSCLC. Pathology, histology, the presence or absence of gene mutations, and the timing of when this information is known (results of gene-mutation testing) are paramount in the treatment of NSCLC. Other concerns are patient age, performance status, hepatic and renal function, comorbidities, and whether the patient has started systemic (chemotherapy) treatment.

The National Comprehensive Cancer Network (NCCN) has established guidelines for the treatment of NSCLC. Patients with untreated (i.e., have not started first-line chemotherapy), advanced-stage NSCLC with a sensitizing *EGFR* mutation (known prior to the initiation of treatment) should start treatment with an oral EFGR TKI (afatinib [Gilotrif], erlotinib, or gefitinib; NCCN, 2016). For patients whose mutation status (sensitizing *EGFR* mutation) is known after they have started chemotherapy, the NCCN recommends either completing the planned number of chemotherapy treatments or interrupting treatment (chemotherapy) and starting afatinib, erlotinib or gefitinib (NCCN, 2016).

The NCCN has recommendations for patients with disease progression on afatinib or erlotinib. If the patient has oligometastatic disease (one site of metastasis), continue the oral EGFR TKI and give local treatment (e.g., radiation therapy) for the metastasis (NCCN, 2016). If the patient develops widespread metastatic disease, discontinue the oral EGFR TKI and start chemotherapy (NCCN, 2016).

**Oral EGFR TKIs**

*Afatinib*: Afatinib is an oral irreversible ErbB family blocker that blocks signaling from EGFR (ErbB1), EGFR 2 (HER2/ErbB2), EGFR 4 (ErbB4), and transphorylation of ErbB3 (Sequist et al., 2013; Solca et al., 2012). Afatinib was approved by the US Food and Drug Administration (FDA) in July 2013 for first-line treatment of patients with metastatic NSCLC whose tumors express *EGFR* mutations with exon 19 deletions or exon 21 (L858R; National Cancer Institute [NCI], 2013a). The FDA’s approval is based on the results of the LUX-Lung 3 randomization clinical trial of afatinib vs. cisplatin plus pemetrexed (Alimta) in patients with advanced-stage, *EGFR*-mutant adenocarcinoma of the lung (NCI, 2013a).

*Erlotinib*: Erlotinib is a potent and selective oral EGFR TKI that reduces HER1/EGFR autophosphorylation, inhibits epidermal growth factor–dependent cell proliferation, and blocks cell-cycle progression at the G1 phase (Perez-Soler et al., 2004; Pollack et al., 1999). Erlotinib has three FDA indications for NSCLC. In 2004, erlotinib was approved by the FDA for second-line treatment of NSCLC, regardless of mutation status, based on results showing improvement in progression-free (PFS) and overall survival (OS), from a phase III clinical trial (Shepherd et al., 2013; Kreamer et al., 2011). The next FDA approval for erlotinib was in April 2010, for maintenance therapy for patients with stable disease after chemotherapy (Cappuzzo et al., 2010). In 2013, erlotinib was approved for first-line treatment of patients with NSCLC with EGFR exon 19 deletions or exon 21 (L858R) substitutions (NCI, 2013b). The first-line treatment approval was based on results of the phase III OPTIMAL randomization clinical trial of erlotinib vs. carboplatin/gemcitabine, which demonstrated improved PFS for patients who received erlotinib (Zhou et al., 2011).

*Gefitinib*: Gefitinib (Iressa) is another oral EGFR TKI that was "re-approved" by the FDA for first-line treatment of NSCLC with EGFR mutations in exon 19 deletions or exon 21 L858R substitution gene mutations (FDA, 2015b). This new approval is based on results from phase III and IV clinical trials (Mok et al., 2008; Douillard et al., 2014), showing improved objective response rates and PFS in patients with sensitizing *EGFR* mutations treated with gefitinib.

**EGFR Resistance**

Patients treated with oral EGFR TKIs eventually develop treatment resistance to these drugs (Gibbons & Byers, 2014; da Cunha Santos et al., 2011). Repeat biopsies of tumors (from patients with initial sensitizing *EGFR* mutations) have identified secondary mutations including T790M, present in approximately 50% of *EGFR*-mutated patients (da Cunha Santos et al., 2011; Gibbons & Byers, 2014). The T790M mutation prevents binding of the TKI to the intracellular domain (Gibbons & Byers, 2014). Other mechanisms of drug resistance include bypass mechanisms, such as MET overexpression, alterations in other HER family proteins, downstream activations of the RAS or PI3K pathways, and transformation from NSCLC to small cell lung cancer (SCLC; Gibbons & Byers, 2014).

The presence of the T790M mutation previously posed treatment challenges for an oncology team. The treatment options for patients with the T790M mutation were discontinuation of the oral EFGR TKI drug and start chemotherapy; cetuximab (Erbitux) with afatinib; or, if eligible, participation in clinical trials with drugs that overcome the T790M mutation. There is an FDA approved agent, osimertinib [Tagrisso], specifically for patients with the T790M mutation (FDA, 2015d).

The combination of cetuximab and afatinib is an effective, yet toxic, treatment for patients with the T790M mutation (Gibbons & Byers, 2014; Jänne et al., 2015), based on results of a phase I clinical trial by Janjigian and colleagues (2014). The overall response rate is 29%, 32% for patients with the T790M mutation, and 25% for patients without the T790M mutation (Janjigian et al., 2014). The median PFS is 4.7 months, and the median duration of confirmed overall response is 5.7 months (Janjigian et al., 2014; Gibbons & Byers, 2014). The combination of cetuximab and afatinib is associated with high rates of toxicities, especially skin and gastrointestinal (Janjigian et al., 2014; Gibbons & Byers, 2014).

Two oral drugs, osimertinib and rociletinib (CO-1686), were evaluated in clinical trials for patients with T790M mutations. Results from the clinical trial by Jänne and colleagues (2015) demonstrated osimertinib has activity in patients with the T790M mutation, previously treated with an oral EGFR TKI. The response rate was 61% for evaluable patients with confirmed T790M mutation; 21% for evaluable patients without the T790M mutation (Jänne et al., 2015). The median PFS is 9.6 months for T790M-positive patients vs. 2.8 months for patients without the mutation (Jänne et al., 2015). In November 2015, the FDA approved osimertinib for EGFR-positive patients with T790M mutation who have disease progression on an oral TKI (FDA, 2015d). The most common side effects were rash, diarrhea, nausea, and decreased appetite (Jänne et al., 2015). The NCCN guidelines (2016) include osimertinib as a second-line treatment option for patients with the T790M mutation who have progressed on previous TKIs.

Rociletinib was another oral TKI agent targeting the T790M mutation. Results from a phase I/II clinical trial evaluating rociletinib demonstrated activity in patients with the T790M mutation (Sequist et al., 2015). Rociletinib has not been granted new drug approval by the FDA as updated data revealed lower response rates than initially reported (Broderick, 2016).

## KRAS

In the United States, activating *KRAS* mutations are found in approximately 20% to 25% of patients with adenocarcinoma of the lungs (both white and African American) and are generally associated with smoking (Sequist & Neal, 2015; Cote et al., 2011; Reinersman et al., 2011). The RAS family of proteins is a central mediator of the mitogen-activated protein kinase (MAPK); signal transducer and activator of transcription (STAT); and phosphoinositide 3-kinase (PI3K) signaling pathways, which together control cell proliferation and apoptosis (Sequist & Neal, 2015). Oncogenic RAS mutations, most commonly those that correspond to missense substitutions in codons 12, 13, and 61, cause continual activity of RAS independent of upstream signals (Sequist & Neal, 2015).

There are conflicting data regarding the presence of the *KRAS* mutation and response or resistance to certain therapies (Sequist & Neal, 2015; Kreamer et al., 2011). In the TRIBUTE clinical trial, a phase III randomization study comparing first-line treatment of platinum-based doublet chemotherapy with/without erlotinib, 55 patients (21%) tested positive for *KRAS* mutation (Sequist & Neal, 2015; Kreamer et al., 2011). The patients with *KRAS* mutation who received chemotherapy (carboplatin/paclitaxel) alone (without erlotinib) had a response rate of 23%, whereas the *KRAS* mutation–positive patients who received chemotherapy and erlotinib had a response rate of 8% as well as worse overall survival (Eberhard et al., 2005).

There is a suggestion that *KRAS* mutations may sensitize tumors to antifolates, such as pemetrexed, possibly by upregulation of a microRNA (mir-181c), which downregulates KRAS (Sequist & Neal, 2015). In a combined analysis of four adjuvant chemotherapy trials, patients with KRAS codon 12 mutation similarly benefitted from chemotherapy as patients with wild-type KRAS; however, the presence of codon 13 mutation appeared to be predictive of worse survival from adjuvant chemotherapy, although the sample size was small (Shepherd et al., 2013).

**Treatment of *KRAS*-Mutant Disease**

Treatment of *KRAS* mutation–positive patients remains a challenge. Multiple early efforts to identify specific RAS inhibitors that are clinically useful against *KRAS*-mutated lung cancer were unsuccessful (Sequist & Neal, 2015). Presently, the focus/target for treating *KRAS*-mutated lung cancers is against downstream effectors of activated KRAS (Sequist & Neal, 2015).

In a phase II clinical trial, 87 patients with previously treated KRAS-mutant NSCLC were randomized to receive treatment of docetaxel with selumetinib (an oral MEK inhibitor) vs. docetaxel with placebo; results from this study showed improved PFS in the selumetinib arm (5.3 months) compared with the placebo are (2.1 months; Jänne et al., 2013). There was also a trend toward improved OS of 9.4 months vs. 5.2 months in the combination arm of docetaxel plus selumetinib vs. docetaxel plus placebo (Jänne et al., 2013). Objective partial responses were seen in 16 of 43 patients (37%) treated with docetaxel plus selumetinib vs. none of 40 in the docetaxel plus placebo arm; however, there was also greater toxicity (more febrile neutropenia, diarrhea, nausea, vomiting, rash) in the docetaxel plus selumetinib arm (Jänne et al., 2013).

Other phase II clinical trials have looked at combinations of MEK inhibitors with oral EGFR TKIs and also other MEK inhibitors. One phase II randomization clinical trial evaluated selumetinib vs. selumetinib plus erlotinib. The result of this study indicated that there was no increased activity with the combination arm in patients with wild-type or *KRAS*-mutant NSCLC (Carter et al., 2013).

Trametinib (Mekinist), an oral MEK inhibitor FDA approved for treatment of melanoma, may have some activity in NSCLC, based on results of phase II clinical trials (Flaherty et al., 2012; Gandara et al., 2013; Blumenschein et al., 2015). More research is needed in this area of treating KRAS-mutant NSCLC. Currently, the treatment recommendations are for patients with *KRAS*-mutant NSCLC to receive standard-of-care chemotherapy or, if eligible, to participate in a clinical trial (Sequist & Neal, 2015).

## ANAPLASTIC LYMPHOMA KINASE

Anaplastic lymphoma kinase is another driver mutation found in a small percentage (approximately 3% to 7%) of patients with NSCLC and in a smaller percentage of African American patients (approximately 1.7%; Pao & Girard, 2011; Soda et al., 2007; Choi et al., 2008; Koivunen et al., 2008; Horn & Pao, 2009; Araujo et al., 2015). Tumors that are ALK-positive contain the fusion of echinoderm microtubule-associated protein-like 4 gene and the *ALK* gene (EML4-ALK), which causes constitutive kinase activity; this activity is associated with uncontrolled cell growth and proliferation (Shaw & Solomon, 2014; Soda et al., 2007). The ALK protein is a receptor kinase in the insulin receptor superfamily (Luo & Lam, 2013).

Clinical features associated with *ALK*-positive tumors include light smoking history or never smokers, younger age of patients, and diagnosis of adenocarcinoma with signet ring or acinar histology (Takahashi et al., 2010). Tumors that are *ALK*-positive are sensitive to treatment with ALK-targeted therapy (Shaw & Solomon, 2014). There are first- and second-generation, FDA-approved treatments for patients with *ALK*-positive NSCLC.

**Treatment of *ALK*-Positive NSCLC**

Treatment recommendations from the NCCN for patients with *ALK*-positive NSCLC mirror those for *EGFR*-mutant NSCLC: If the tumor is *ALK*-positive and the patient has not started systemic treatment, begin treatment with an oral targeted therapy (NCCN, 2016). If results of mutation testing are known after starting systemic treatment (chemotherapy), stop chemotherapy and begin targeted treatment or complete the prescribed number of chemotherapy treatments and then begin treatment with targeted therapy (NCCN, 2016). The NCCN also recommends continuing oral targeted therapy even if the patient develops isolated metastases (one site or multiple metastases in only one site) and consider local treatment (radiation) for the metastases (NCCN, 2016).

*Crizotinib*: Crizotinib (Xalkori) is a multitargeted small molecule TKI and a potent inhibitor of ALK phosphorylation and signal transduction (Christensen et al., 2007). Crizotinib induces rapid tumor regression and objective responses in the majority of patients with *ALK*-positive tumors (Shaw et al., 2013).

Results from a phase III randomized clinical trial of crizotinib vs. single-agent chemotherapy (pemetrexed or docetaxel) for previously treated patients showed increased PFS for patients randomized to treatment with crizotinib (the median PFS was 7.7 months vs. 3 months) compared with those treated with chemotherapy (Shaw et al., 2013). Responses were achieved more rapidly on the crizotinib arm (6.3 weeks) than the chemotherapy arm (12.6 weeks) and were of longer duration (32 weeks vs. 24 weeks; Shaw et al., 2013).

Results from another clinical trial that randomized untreated (no systemic treatment) patients to crizotinib vs. platinum-based chemotherapy showed improved PFS for the crizotinib arm (10.9 months) vs. the chemotherapy arm (7 months) and a higher overall response rate (74%, crizotinib; 45%, chemotherapy; Mok et al., 2009).

*Resistance to ALK Inhibitors*: Patients treated with crizotinib eventually develop resistance to the drug. Causes of resistance include tumor acquisition of a secondary mutation within the ALK tyrosine kinase domain, amplification of the *ALK* fusion gene (which may occur alone or in combination with a secondary resistance mutation), or development of alternative or bypass signaling pathways (Shaw & Solomon, 2014).

The most common resistance mutations are the gatekeeper L1196M mutation and G1296A (Shaw & Solomon, 2014). One notable mutation is G1202R, as it confers high-level resistance to crizotinib and next-generation ALK inhibitors (Shaw & Solomon, 2014).

Another mode of crizotinib resistance is the development of alternative or "detour" signaling pathways, including abnormalities in EGFR, KIT, and insulin-like growth factor-1 receptor (IGFR-1) pathways (Shaw & Solomon, 2014). This last method of crizotinib resistance suggests, perhaps, evaluating combination (targeted) therapies to overcome resistance (Shaw & Solomon, 2014).

*Ceritinb*: Ceritinib (Zykadia) is a second-generation, FDA-approved ALK inhibitor for patients who are or have become resistant to crizotinib (Shaw & Solomon, 2014). Ceritinib is more potent than crizotinib (Shaw & Solomon, 2014). Based on results of clinical trials, the FDA approved ceritinib in April 2014 for patients whose tumors had progressed on, or were intolerant to, crizotinib (Shaw & Solomon, 2014).

Currently, there are clinical trials looking at other second-generation ALK-targeted therapies, such as alectinib (Alecensa; Shaw & Solomon, 2014). For patients with *ALK*-positive NSCLC, there are two FDA-approved drugs and ongoing clinical trials for other agents.

*Alectinib*: Alectinib is another second-generation ALK inhibitor with activity in crizotinib-resistant disease and brain metastases (Shaw & Solomon, 2014; Seto et al., 2013; Gadgeel et al., 2014; Gainor et al., 2015; Ou et al., 2015). Outcomes from the phase I/II clinical trial that evaluated the safety and activity of alectinib in patients with crizotinib-resistant NSCLC and brain metastases demonstrated alectinib’s efficacy in treating brain metastases (Gadgeel et al., 2014). Additionally, analysis of cerebral spinal fluid (CSF) from five patients showed drug concentrations of alectinib in the CSF (Gadgeel et al., 2014).

Findings from a phase II single-arm clinical trial confirmed alectinib has activity in treating brain metastases (Ou et al., 2015). In November 2015, the FDA approved alectinib for treatment of patients with *ALK*-positive NSCLC who progressed on or are intolerant of crizotinib (FDA, 2015c). The NCCN guidelines recommend either ceritinib or alectinib as second-line treatment for patients with ALK-positive NSCLC who progressed on crizotinib (NCCN 2016).

## ROS1

ROS1 is a receptor tyrosine kinase of the insulin receptor family and a potent oncogenic driver (Shaw & Solomon, 2014; Gainor & Shaw, 2013). ROS1 rearrangements are believed to promote signal transduction leading to upregulation and activation of various intracellular pathways, resulting in promotion of cell survival and proliferation (Gainor & Shaw, 2013). ROS1 rearrangements have been found in 1% to 2% of patients with NSCLC and were associated with younger-age, never smokers, Asian ethnicity, and, advanced stage (Gainor & Shaw, 2013; Bergethon et al., 2012). The predominant histology for ROS1-positive NSCLC is adenocarcinoma, although ROS1 has been found (infrequently) in large cell and squamous cell histologies (Gainor & Shaw, 2013; Davies et al., 2012; Rimkunas et al., 2012).

Results from a phase I clinical trial showed that crizotinib had activity, with objective responses, duration of response, and PFS, in patients with ROS 1 positive NSCLC (Shaw et al., 2014). In March 2016, the FDA approved crizotinib for the treatment of *ROS1*-positive tumors (Briz, 2016).

## IMMUNOTHERAPY

**Programmed Cell Death Protein 1 (PD-1)/Programmed Cell Death Ligand 1 (PD-L1)**

Immunotherapy is the newest treatment modality for NSCLC. The goals of immunotherapy for cancer are aiding the immune system to recognize cancer (cells) as foreign bodies, stimulate immune responsiveness, and relieve the inhibition of the immune system that allows for tumor growth (Gettinger, 2015).

Generating an effective antitumor immune response is a complex multistep process (Chen, Irving, & Hodi, 2012). First, the T cells (of the immune system) must be able to recognize cancer cells as foreign and then generate cytotoxic T lymphocytes (CTLs) to travel to and infiltrate tumors to bind to the cancer cells and kill them (Chen et al., 2012). Each step of the process must happen to derive clinical benefit (Chen et al., 2012).

Research and clinical data have shown the importance of one inhibitory ligand and receptor pair—PD-L1 and PD-1—in inhibiting the last step in the process: preventing the killing of cancer cells by CTLs (Chen et al., 2012). Tumors that express PD-L1 are able to inactivate the normal immune system’s response to killing cancer cells. Cytotoxic T lymphocytes become nonfunctional by engaging the inhibitory receptor PD-1 (Chen et al., 2012).

Expressed on the surface of T cells, PD-1, when activated, binds to PD-L1, triggering an inhibitory signal that results in reduced cytokine production and (reduced) proliferation of T cells (Carter et al., 2002; Freeman et al., 2000). And PD-L1 is upregulated in tumors through activation of key oncogenic pathways (PI3K, MAPK; Chen et al., 2012). It is through upregulation of PD-L1 expression that cancer cells evade detection by the host immune system and progress (Chen et al., 2012).

**Treatment Targeting PD-L1**

Antibodies that target PD-L1 act mainly by inhibiting the binding of PD-L1 to PD-1, thus freeing cancer-specific CTLs to mediate killing of cancer cells that express PD-L1 (Pardoll, 2012; Mellman & Nelson, 2008). There are now two FDA-approved monoclonal antibodies for treatment of NSCLC that target PD-1: nivolumab (Opdivo) and pembrolizumab (Keytruda; Sosman, 2015).

*Nivolumab*: Based on the results of two clinical trials, nivolumab received FDA approval (in March 2015) for treatment of NSCLC in patients with advanced squamous cell carcinoma previously treated with chemotherapy who had disease progression on or after treatment with platinum-based chemotherapy The results of the phase III randomized CheckMate 017 trial, nivolumab vs. docetaxel in previously treated patients with squamous cell carcinoma showed improved median survival of 9.3 months for the patients treated with nivolumab vs. 6 months for patients treated with docetaxel (Brahmer et al., 2015). The phase II single-arm clinical trial (CheckMate 063) showed nivolumab had activity (in terms of response and survival) in previously treated patients with squamous cell histology after at least two prior lines of chemotherapy (Rizvi et al., 2015).

In March 2015, the FDA approved nivolumab for the treatment of patients with advanced squamous cell carcinoma of the lungs (Gettinger, 2015). Results from the phase II single-arm CheckMate 063 study showed nivolumab had activity (in terms of response and survival) in previously treated patients with squamous cell histology (Rizvi et al., 2015). Outcomes from the phase III randomized CheckMate 017 study nivolumab vs. docetaxel revealed a survival benefit for the patients who received nivolumab (9.3 months vs. 6 months; Brahmer et al., 2015).

*Pembrolizumab*: Pembrolizumab is another monoclonal antibody approved by the FDA for treatment of NSCLC. Based on the results of a phase I clinical trial, pembrolizumab received breakthrough therapy designation for advanced NSCLC in late 2014 (Gettinger, 2015). Results of this clinical trial revealed a median duration of response of 12.5 months, PFS of 3.7 months, and OS of 12 months (Garon et al., 2015). Recently, the FDA approved pembrolizumab for treatment of NSCLC (FDA, 2015a). The 2016 NCCN guidelines recommend either nivolumab or pembrolizumab as preferred subsequent treatment for metastatic NSCLC (NCCN, 2016).

## CONCLUSION

For many decades, the prognosis for advanced-stage NSCLC was not bright. Chemotherapy, the mainstay of treatment, had limited benefit and much toxicity. Now with mutation testing and appropriate treatments, there has been a trend toward improved PFS and OS. There is an array of treatments for mutant-positive NSCLC, with research evaluating newer generations of targeted therapies. Additionally, immunotherapy is now part of the treatment armament. The treatment horizon for advanced-stage NSCLC is greatly expanded, with more options available to patients and their oncology team.
